# Peripheral Macrophages Promote Tissue Regeneration in Zebrafish by Fine-Tuning the Inflammatory Response

**DOI:** 10.3389/fimmu.2019.00253

**Published:** 2019-02-15

**Authors:** Rodrigo A. Morales, Miguel L. Allende

**Affiliations:** FONDAP Center for Genome Regulation, Facultad de Ciencias, Universidad de Chile, Santiago, Chile

**Keywords:** macrophage, tissue-resident, migration, regeneration, *csf1ra*, *il1b*, ROS

## Abstract

The role of macrophages during regeneration in zebrafish has been well-documented. Nevertheless, new evidence indicates that zebrafish macrophages are a heterogeneous population of cells, and that they can play different roles during immune responses and in tissue restoration after damage and infection. In this work, we first aimed to classify zebrafish macrophages according to their distribution in the larva during homeostasis and after tissue damage, distinguishing peripheral, and hematopoietic tissue resident macrophages. We discovered differences between the migratory behavior of these two macrophage populations both before and after tissue damage, triggered by the amputation of the tail fin. Further, we found a specific role for peripheral tissue-resident macrophages, and we propose that these cells contribute to tail fin regeneration by down-regulating inflammatory mediators such as interleukin-1b (*il1b*) and by diminishing reactive oxygen species (ROS) in the damage site. Our work suggests that specific macrophage populations recruited after tissue damage in zebrafish larvae can display different functions during both inflammation and tissue regeneration.

## Introduction

Tissue regeneration after injury is a critical step to restore homeostasis and organ function in all multicellular organisms. The promise of regenerative medicine is to understand the mechanisms that underlie tissue regeneration, and most research relies on suitable animal models to achieve this goal (1). For many years, the zebrafish has been used as a model in this field because of its strong regenerative capacity in organs and tissues such as the heart, retina, peripheral, and central nerves, fins, among others (2). Tail fin regeneration is a conserved, robust, and highly reproducible model of epimorphic regeneration, which is characterized by the formation of a blastema, a group of stromal cells with a high proliferative capacity and differentiation ability, that can grow and regenerate the lost limb completely (3, 4). Although this model was initially described in adult zebrafish, it was demonstrated that the regeneration of the tail fin in zebrafish larvae after amputation is similar to adults, sharing blastema formation, and complete regeneration of the tail fin a few days after amputation (5).

In recent years, attention has been directed toward understanding the contribution of the immune system to tissue regeneration, particularly, the role of macrophages (6, 7). These phagocytic cells display different functions during the immune response, contributing with proinflammatory signaling during the early phases of inflammation, and promoting tissue repair and regeneration in late stages, once inflammation is resolved (8, 9). The relationship between macrophages and tissue regeneration has been documented in zebrafish, as macrophages were shown to be essential for caudal fin regeneration in adults and larvae (10, 11), and are a key player in zebrafish cardiac regeneration as well as in medaka (12). However, it has been described that macrophages are not a homogeneous population of cells and they can be differentially classified according to their function as well as with respect to their physical location in the organism (13, 14). These findings have benefitted from the use of fluorescent reporter lines, which allow the tracking of different cell types, including macrophages, in the transparent larvae (15). In 72 h post fertilization (hpf) zebrafish larvae, macrophages can be found distributed in several peripheral tissues such as the brain, heart, retina, and muscle. However, there is also a concentration of macrophages in a transient hematopoietic tissue, the caudal hematopoietic tissue (CHT). Macrophages have been depleted from zebrafish using different strategies including morpholino knockdown, genetic ablation, and mutation (10, 16–18), but little is known about the specific roles of different types of macrophages during regeneration. It was demonstrated recently that macrophages can polarize from an M1-like to an M2-like phenotype after tissue damage (19), and that the M2-like cells can promote tail fin fold regeneration after amputation (11), thus emphasizing that larval zebrafish macrophages are a functionally heterogeneous population of cells.

In this work, we aimed to find functional differences between macrophages according to their location, distinguishing peripheral tissue-resident from CHT-derived cells. We also aimed to determine if cells belonging to either or both classes contribute to tail fin regeneration in zebrafish larvae. We found that peripheral and CHT-derived macrophages show different behaviors both during homeostasis and after tail fin amputation. Using specific genetic or chemical cell ablation methods, we also show that peripheral tissue-resident macrophages are the main contributors of the tail fin regeneration in zebrafish larvae, a role possibly mediated by *il1b* and ROS down-regulation. Our results contribute to the growing body of evidence that reveals a heterogeneity in the roles of macrophages in physiology and regeneration in teleosts.

## Materials and Methods

### Fish Husbandry and Lines

Zebrafish (*Danio rerio*) were reared and kept in our facilities according to standard procedures (20). The fish lines used in this work were the *csf1ra*^*j*4*blue*^ (*panther*) mutant (21); *Tg(mpeg1:Dendra2)* (22) and *TgBAC(mpx:GFP)*^*i*114^ (23). Homozygous *csf1ra*^*j*4*blue*^ adults are viable and were identified and sorted from their phenotypically wild type siblings by their pigmentation, because of the lack of xantophores (21), and they were incrossed to obtain homozygous mutant larvae. All procedures complied with the “Guidelines for the Use of Fishes in Research Use” of the American Fisheries Society (Guidelines for the use of fishes in research. American Fisheries Society, Bethesda, Maryland. www.fisheries.org) and were approved by the Animal Ethics Committee of the University of Chile (document approval: 2015-04-20).

### Steady State Time Lapse Imaging

At 3 days post fertilization or dpf (72 h post fertilization or hpf), transgenic *Tg(mpeg1:Dendra2)* larvae were mounted in 0.8% low melting point agarose solution with 0.01% MS-222 (Sigma, St. Louis, MO, USA). Time lapse imaging of a portion of the tail, considered as the section of the larvae posterior to the anus, were performed using an Olympus IX81 epifluorescence microscope (Olympus, Tokyo, Japan) with a 10x zoom, every 2 min for a total of 3 h. The average speed for the imaged macrophages was calculated using the Manual Tracking plugin in the ImageJ software (NIH, Bethesda, ML, USA).

### Tail Fin Amputation and Macrophage Quantification

**Figure 1:**
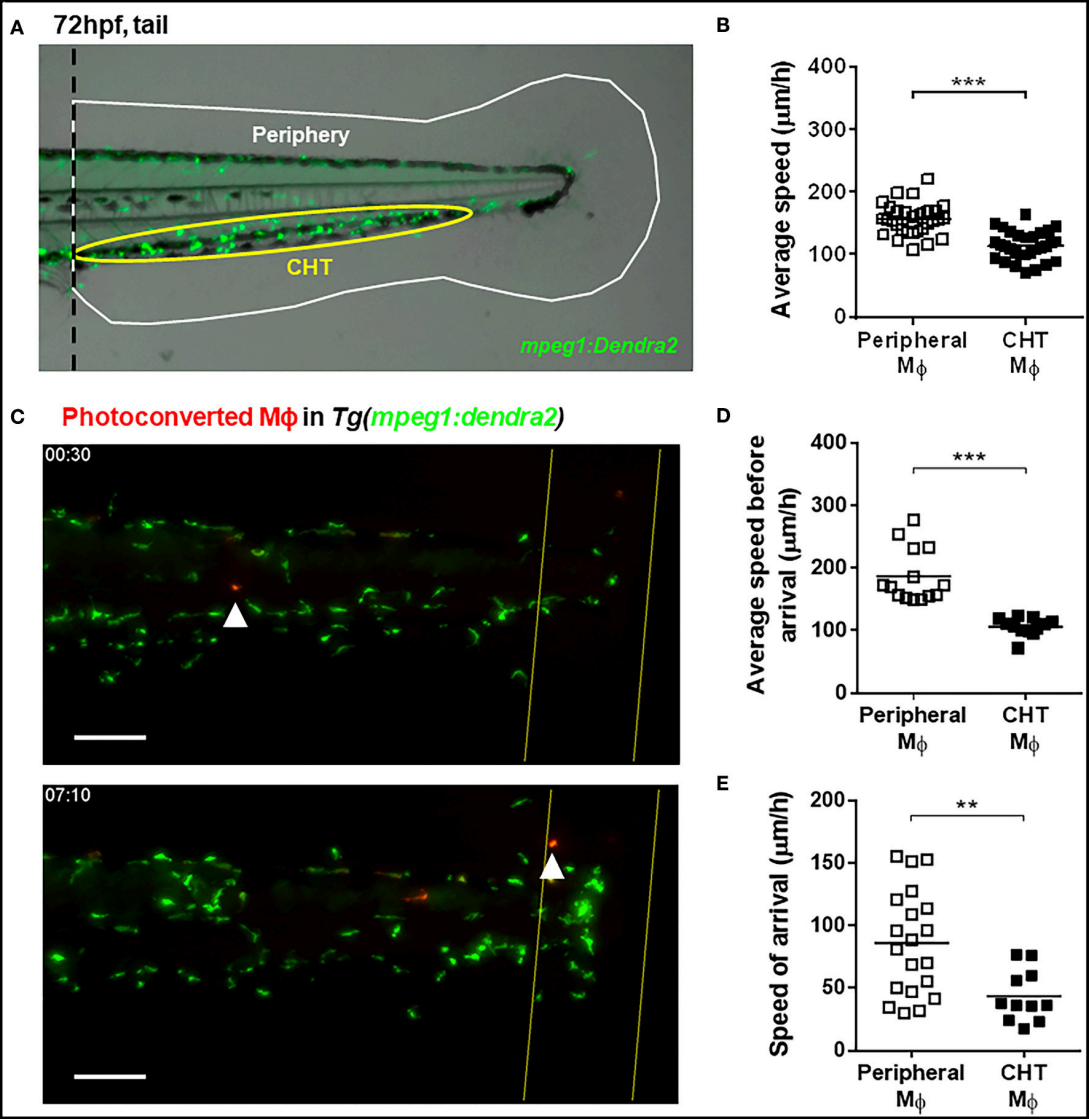
Kinetic differences between peripheral tissue-resident and CHT-resident macrophages in steady state and after damage. **(A)** At 72 hpf, macrophages in the *Tg(mpeg1:Dendra2)* reporter line were classified as peripheral tissue-resident macrophages (white area) or CHT-resident macrophages (yellow area). **(B)** Average speed of peripheral and CHT macrophages (Mϕ) in steady state conditions. A tail portion of *Tg(mpeg1:Dendra2)* larva was imaged every 3 min for 3 h. **(C)** Time lapse imaging of photoconverted macrophages recruited to the damaged site in *Tg(mpeg1:Dendra2)* larvae. The photoconversion was performed before damage, and the complete tail region was captured every 5 min for a total of 24 h. Representative images showing a peripheral macrophage (white arrowhead) in the start of the time lapse (upper image), and at the time of recruitment to the damage site (lower image). Scale bar = 50 μm. **(D)** The average speed and **(E)** the speed of arrival of individual photoconverted macrophages. A total of 14 peripheral macrophages and 12 CHT macrophages from a pool of photoconverted individuals were used for the analysis. ^**^p < 0.01; ^***^p < 0.001.

For tail fin amputation, 3 dpf larvae were anesthetized with MS-222 and amputated with a sterile scalpel. The transection was performed by using the posterior section of the ventral pigmentation gap in the tail fin as a reference, and immediately after amputation larvae were rinsed and incubated in E3 medium at 28°C. Images of recruited neutrophils and macrophages to the damage site (up to ~150 μm from the amputation site) were captured using an Olympus MVX10 stereomicroscope or an Olympus IX81 epifluorescence microscope, and analyzed using ImageJ software. The quantification of peripheral and CHT macrophages in non-amputated larvae was performed according to their location in the tail (Figure 1A). For the normalization of recruited macrophages, the number of recruited macrophages was divided by the total number of macrophages located in the tail, i.e., the sum of peripheral macrophages, CHT macrophages, and recruited macrophages.

### Photoconversion of Dendra2-Labeled Macrophages and Time Lapse After Damage

At 3 dpf and before tail fin amputation, groups of macrophages in fish harboring the photoconvertible reporter *Tg(mpeg1:Dendra2)* localized at a distance between 400 and 600 μm from the transection line were photoconverted from green to red using a Zeiss Axiovert 200 M fluorescence microscope (Carl Zeiss, Jena, Germany) equipped with a Mercury lamp. The photoconversion was performed exposing a portion of the tail to a 385 nm laser for 40 s, using a 40x zoom. After photoconversion, individuals were amputated and immediately mounted in 0.8% low melting point agarose solution with 0.01% MS-222. Time lapse of the whole larval tail was performed in the Olympus IX81 microscope equipped with a 4x zoom, starting from 30 min after amputation and imaging every 5 min for a period of 24 h. Data analyses, including the average speed of photoconverted macrophages, the calculation of its initial distance and the time of their arrival to the damage site was performed using ImageJ.

### Clodronate Liposome Injection

For partial macrophage depletion, a dilution of clodronate liposomes (henceforth referred to as lipo-clodronate) (24, 25) was injected in the *circulation valley* of 54 hpf zebrafish larvae, thus allowing the spread of lipo-clodronate in the whole larva through the bloodstream. Control larvae were injected with a same dilution of PBS-loaded liposomes (lipo-PBS). Eighteen hours post injection (72 hpf or 3 dpf), larvae were sorted and amputated.

### Quantification of Tail Fin Regeneration

At 3 and 5 days post amputation or dpa (6 and 8 days post fertilization, respectively), larvae were mounted in 1% low melting point agarose, and regenerating tail fins were imaged in bright field using the Olympus MVX10 microscope, with a 5x zoom. Tail fin area was measured from the anterior section of the ventral tail fin gap to the end of the regenerating fin, as previously described (26), using ImageJ software. Tail fin areas were calculated and expressed in square millimeters (mm^2^).

### BrdU Incorporation and Immunohistochemistry

Amputated larvae were incubated in 5 mM of 5-Bromo-2′-deoxyuridine (BrdU, EMD Chemicals, San Diego, CA, USA) diluted in E3 medium with 1% DMSO, from 6 to 24 h post amputation (hpa). After incubation, larvae were fixed in 4% paraformaldehyde (PFA) and then stored at −20°C in methanol. For BrdU detection, larvae were rinsed several times with PBS-Triton X-100 0.3% (PBS-Tx), then permeabilized with proteinase K 20 μg/mL for 30 min at room temperature, refixed with PFA 4% per 20 min at room temperature and washed with PBS-Tx. To increase permeability, larvae were incubated in cold acetone for 7 min at −20°C, then rinsed with PBS-Tx. For BrdU epitope exposure, samples were incubated in HCl 2N for 20 min at room temperature and then rinsed several times with PBS-Tx. Larvae were incubated in blocking solution (BSA 10 mg/ml, goat serum 2%, DMSO 1%, Triton X-100 0.1% in PBS) for at least 45 min and room temperature, and then incubated with 1:500 of mouse monoclonal anti-Bromodeoxyuridine Bu20a (M0744, Dako, Glostrup, Denmark) overnight at 4°C. After primary antibody incubation and washes with PBS-Tx, samples were incubated with 1:2,000 of Alexa Fluor 488 goat anti-mouse IgG (A11029, Invitrogen, Eugene, OR, USA) for 2 h at room temperature in darkness, and rinsed several times with PBS-Tx. Stained larvae were imaged in a Zeiss LSM 510 Meta confocal Microscope (Carl Zeiss), and BrdU dots were quantified using ImageJ software. As the ventral pigmentation gap area contains a group of proliferating cells, we restricted the quantification of BrdU+ dots to the region immediately adjacent to the damaged site.

### Cell Death Analysis

For cell death quantification, TUNEL analysis was performed in 24 hpa larvae previously fixed in 4% PFA and dehydrated with methanol, using the ApopTag red *in situ* apoptosis detection kit (Merck Millipore, Temecula, CA, USA). Stained larvae were imaged using a confocal microscope and analyzed using ImageJ.

### ROS Detection

For ROS detection in the damage site, 6 hpa larvae were rinsed several times with Calcium-free HBSS medium (Gibco, Carlsbad, CA, USA) and then incubated in 50 μM of the ROS sensor 2′7′-dichlorohydrofluorescein diacetate (H_2_DCF-DA, Sigma) for 15 min at 28°C in darkness. After incubation, larvae were rinsed in E3 medium and mounted in 0.8% low melting point agarose with MS-222. Images were acquired using the Olympus MVX10 stereomicroscope, with a 6.3x zoom. Fluorescence quantifications were obtained using ImageJ, and the relative fluorescence in the damage site was calculated as the ratio between the fluorescence in the damage site by the fluorescence in the tail (~500 μm far from the damage site) of the same individual.

### Quantitative RT-PCR

After 6 and 24 hpa, *panther* and wild-type larvae were euthanized by MS-222 overdose, larval tails were collected, and RNA was isolated using the RNAqueous-Micro kit (Ambion, Lithuania). cDNA from isolated RNA samples were generated using ImProm-II Reverse Transcription system (Promega, Madison, WI, USA), and qPCR was performed in a Stratagene Mx3000P system using Brilliant II SYBR Green as fluorescent detector (both from Agilent Technologies, Cedar Creek, TX, USA). Primers used were: *il1b* Fwd 5′-TGGACTTCGCAGCACAAAATG-3′ and Rev 5′-CGTTCACTTCACGCTCTTGGATG-3′ (27); *tnfa* Fwd 5′-CAAGGCTGCCATCCATTTAACAGG-3′ and Rev 5′-TCAGTTCAGACGTGCAGCTGAT-3′; *tgfb1a* Fwd: 5′- CAACCGCTGGCTCTCATTTGA-3′ and Rev 5′- ACAGTCGCAGTATAACCTCAGCT-3′ (19); *il10* Fwd 5′- ACAGTCCCTATGGATGTCACGTCA-3′ and Rev 5′- GCATTTCACCATATCCCGCTTGAG-3′; *ef1a* Fwd 5′-AGAAGGAAGCCGCTGAGATG-3′ and Rev 5′-TGTCCAGGGGCATCAATAAT-3′ (27). For analysis, target gene expression was calculated using the 2^−Δ*Ct*^ method (28), using *ef1a* as a reference housekeeping gene.

### Statistical Analyses

Student's *t*-tests were performed when comparing two conditions, whereas two-way ANOVA with a Tukey post-test were used to analyze grouped data. Both statistical tests were performed using GraphPad Prism 6.0 software (GraphPad, San Diego, CA, USA).

### Online Supplemental Material

Supplemental materials include: videos of macrophage recruitment in photoconverted reporter larvae (Supplemental Video 1); analyses of neutrophils in *panther* (Supplemental Figure S1) and after the injection of a 1:50 dilution of clodronate liposomes (Supplemental Figure S2); quantifications of macrophage number and tail fin regeneration following a 1:10 injection of clodronate liposomes (Supplemental Figure S3).

## Results

### Macrophages Located in Peripheral Tissues and CHT-Derived Macrophages Exhibit Different Behavior During Homeostasis and After Tail fin Amputation

At 72 hpf, zebrafish macrophages can be classified according to their location as CHT-resident or peripheral tissue resident (Figure 1A). We performed time lapse imaging in the tail of 72 hpf *Tg(mpeg1:Dendra2)* zebrafish individuals to compare the spontaneous movement kinetics of CHT-resident and peripheral tissue-resident macrophages in the intact animal. We found that the average speed of peripheral tissue-resident macrophages is higher compared to CHT-resident ones (averages ± standard deviations (SDs) of 155.6 ± 25.3 and 114.2 ± 23.2 μm/h, respectively; Figure 1B), suggesting that both populations may be primed to react in a different fashion when an inflammatory response is triggered. Thus, we sought to analyze whether these populations are recruited in differential fashion to the site of a tissue injury (tail fin amputation). In order to track peripheral and CHT macrophages during damage response, we used the *Tg(mpeg1:Dendra2)* reporter and photoconverted groups of macrophages in either region before damage, allowing us to track them and to analyze their recruitment, and behavior during the inflammatory response triggered by tail fin amputation. We imaged the tail of photoconverted/damaged larvae every 5 min for 24 h, starting at 30 min post amputation (Supplemental Video 1; Figure 1C). In line with the findings under steady state conditions, we observed that the average speed of photoconverted peripheral tissue-resident macrophages is higher compared to photoconverted CHT-resident macrophages (186.8 ± 43.2 vs. 106.3 ± 14.0 μm/h; Figure 1D). Next, we analyzed the migration of peripheral and CHT macrophages to the damage site, analyzing the time of arrival at the damage site for both populations. We found that periphery-derived macrophages are recruited earlier than CHT-derived macrophages (9.7 ± 4.7 and 14.5 ± 3.6 h, respectively; *p* < 0.01). Moreover, when we calculated the speed of travel of macrophages (the ratio between the initial distance of the cells to the site of damage and their time of arrival) we found that peripheral tissue-resident macrophages are recruited faster compared to CHT-resident macrophages (73.5 ± 40.0 vs. 41.3 ± 17.1 μm/h, respectively; Figure 1E). These results indicate that peripheral tissue-resident macrophages and CHT-resident macrophages have different motility in homeostasis and show differences in migration behavior when an inflammatory response is triggered.

### *csf1ra*/*panther* Mutants Exhibit a Strong Reduction in the Peripheral Macrophage Pool and an Altered Recruitment After Tail Fin Amputation

**Figure 2:**
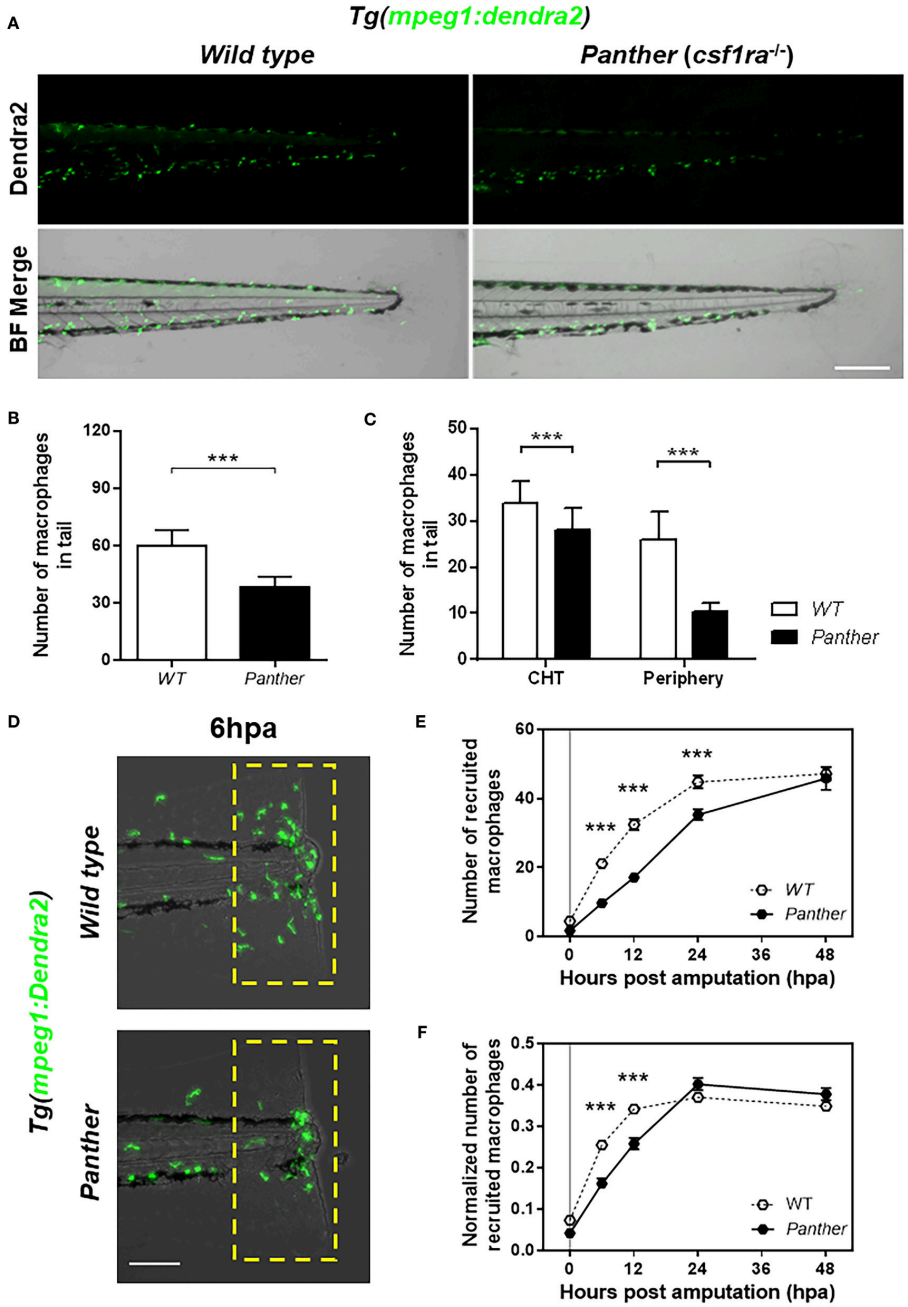
*Panther* (*csf1ra*^−/−^) larvae have a fewer peripheral macrophage population and a delayed recruitment of macrophages after tail fin amputation. **(A)** Representative images of *Tg(mpeg1:Dendra2)* larvae in a wild type (WT) or a *panther* genetic background. Scale bar = 250 μm. **(B)** Total number of macrophages ± *SD* in the tail of *panther* and WT larvae at 72 hpf. **(C)** Quantification of peripheral tissue-resident and CHT-resident macrophages in the tail of *panther* and WT larvae. Means ± *SD*s for each condition are shown in the graph. **(D)** Recruitment of macrophages (green cells in the yellow dashed rectangle) in *panther* and WT individuals after tail fin amputation. Scale bar = 100 μm. **(E)** Quantification of recruited macrophages ± SEM after tail fin amputation in *panther* and WT larvae from 0 to 48 hpa. Twenty larvae per condition were used. **(F)** The previous quantification was normalized by the number of total macrophages in the tail of the respective larva (the sum of peripheral, CHT, and recruited macrophages). ^***^p < 0.001.

It has been previously described that missense mutations in the *colony stimulating factor 1 receptor* gene negatively affect the number of macrophages, most importantly, the peripheral tissue-resident population (29, 30). We used the previously described *csf1ra*^*j*4*blue*^ mutant allele (21), whose homozygotes (referred to from here on as *panther*) exhibit a significant reduction in the pool of tissue-resident macrophages during larval stages (16, 31). We combined the *csf1ra*^*j*4*blue*^ carriers with the macrophage reporter line *Tg(mpeg1:Dendra2)*, to obtain *panther* mutants with Dendra2-labeled macrophages. In the tail of *panther* individuals, we observed a ~40% overall reduction of macrophages compared to wild type animals (Figures 2A,B). A more detailed analysis revealed that *panther* larvae had a reduction in the CHT-resident macrophage population of ~20% compared to wild type, whereas the pool of peripheral tissue-resident macrophages is reduced by ~60% in *panther* compared to wild type fish (Figure 2C). We did not observe differences in the number of neutrophils when we analyzed *panther* mutants in the *TgBAC(mpx:GFP)*^*i*114^ transgenic reporter background (Supplemental Figure S1A). These findings are in line with previous reports (16, 32) and indicate that, although the overall macrophage population is reduced, the pool of peripheral tissue-resident macrophages is more strongly affected in *panther* than the CHT-resident population.

We analyzed the recruitment of macrophages in *panther* mutants after tail fin amputation (Figure 2D) and we found a reduction in the number of macrophages recruited to the damage site from 6 to 24 hpa (Figure 2E). As the decreased recruitment of macrophages could be attributed to the overall reduction of macrophages in *panther* mutants, we divided the number of recruited macrophages of each larva by the total number of macrophages in the tail of the same larva, to obtain a normalized number of recruited macrophages. After this correction, we still found a reduced recruitment of macrophages in *panther* mutants at 6 and 12 hpa, when compared to wild type (Figure 2F). In contrast to macrophages, neutrophil recruitment to the damaged site was indistinguishable between *panther* and wild type individuals (Supplemental Figure S1B). These results support previous findings and further demonstrate that peripheral tissue-resident macrophages are recruited earlier compared to CHT-resident macrophages after tail fin amputation in zebrafish larvae.

**Figure 3:**
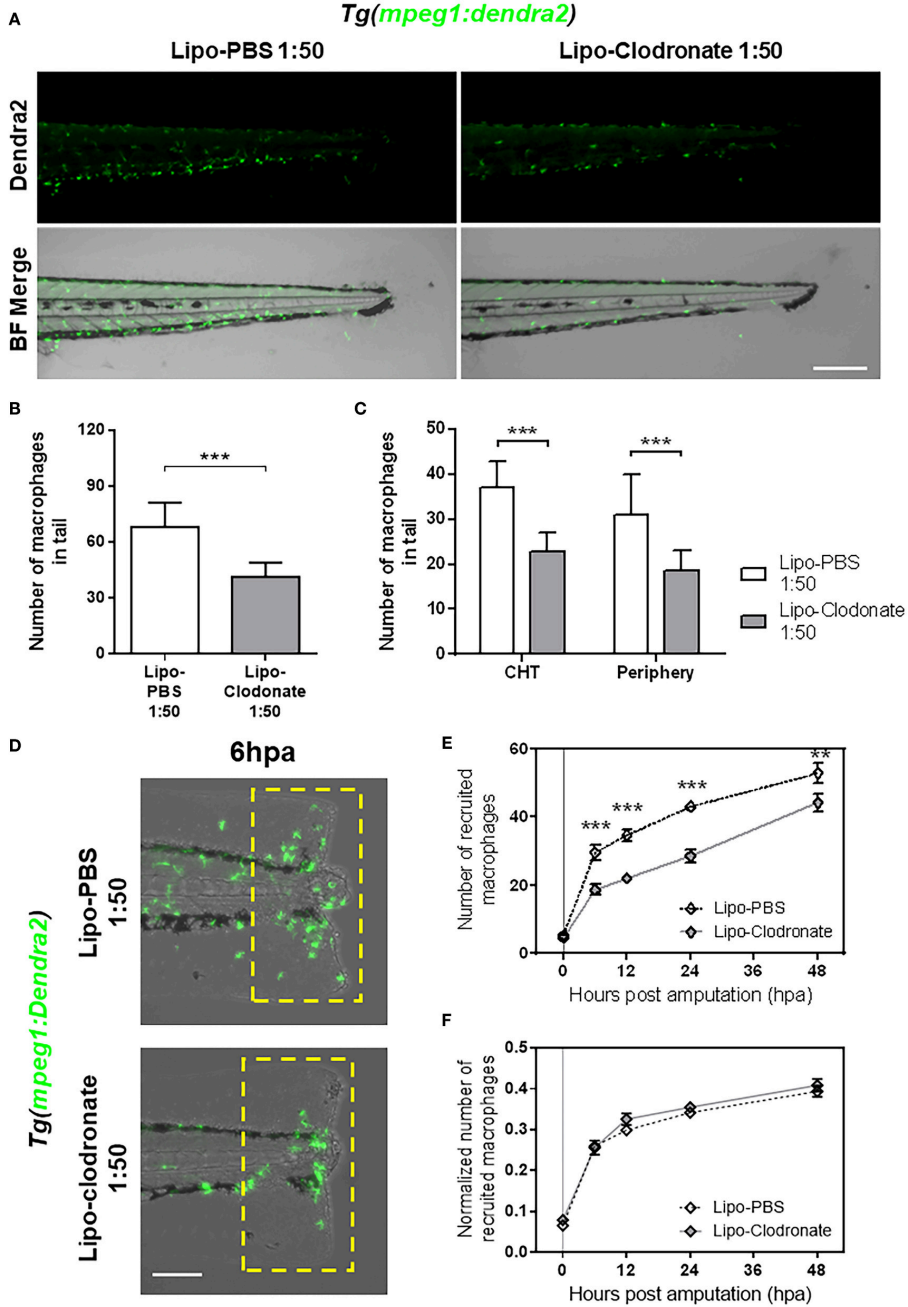
Partial reduction of macrophages pool does not affect the kinetic of macrophage recruitment after tail fin amputation. **(A)** Images of 72 hpf *Tg(mpeg1:Dendra2)* larvae, 18 h after injection of an 1:50 dilution of Lipo-PBS and Lipo-clodronate in the bloodstream, respectively. Scale bar = 250 μm. **(B)** Total macrophages ± *SD* in the tail of Lipo-clodronate 1:50 and Lipo-PBS 1:50 larvae at 72 hpf. **(C)** Mean ± *SD* of peripheral tissue-resident and CHT-resident macrophages in the tail. **(D)** Recruited macrophages (green cells in the yellow dashed rectangle) in Lipo-clodronate 1:50 and Lipo-PBS 1:50 individuals after tail fin amputation. Scale bar = 100 μm. **(E)** Quantification of recruited macrophages ± SEM after tail fin amputation in Lipo-clodronate 1:50 and Lipo-PBS 1:50 larvae from 0 to 48 hpa. A total of 20 larvae per condition were used. **(F)** Normalized number of recruited macrophages at each time point. ^**^p < 0.01; ^***^p < 0.001.

As we found impaired recruitment of macrophages in *panther* mutants, we wanted to determine if this observation could be replicated by reducing the global macrophage pool in zebrafish larvae or if the effect was specific to *panther* mutants. To this end, we used different concentrations of clodronate liposomes to reduce the total macrophage number in larvae to a level comparable with those in *panther* mutants. This method has been used to decrease macrophage number in zebrafish larvae in a local or a systemic fashion (11, 18, 32, 33). We injected lipo-clodronate at 54 hpf in the circulation valley, allowing its distribution throughout the whole larva. At 72 hpf, 18 h after injection of the liposomes, we quantified the number of macrophages in the tail of lipo-clodronate larvae and lipo-PBS controls. We observed that a global reduction of ~40% of macrophages in the tail is achieved when injecting ~5 nl of 1:50 dilution of lipo-clodronate compared to an equal dilution of lipo-PBS (Figures 3A,B). Importantly, when we analyzed the number of CHT-resident and peripheral tissue-resident macrophages, we found a similar reduction in both populations of ~40% (Figure 3C), indicating that the administration of 1:50 lipo-clodronate in circulation affects all macrophages, irrespective of their location in the body. We confirmed that lipo-clodronate injection did not affect neutrophil numbers in zebrafish larvae (Supplemental Figure S2A), as previously reported (18). Next, we analyzed macrophage recruitment after tail fin amputation in 1:50 lipo-clodronate injected larvae at 72 hpf (18 hpi) and we observed a decrease in the number of macrophages recruited at the injured site at all-time points from 6 to 48 hpa (Figures 3D,E). However, these differences went not significant when we normalized all numbers by the number of total macrophages in the tail at their respective timepoints (Figure 3F). As in *panther* larvae, we did not detect differences in neutrophil recruitment after Lipo-clodronate treatment (Supplemental Figure S2B). Thus, our results indicate that the kinetics of macrophage recruitment is not affected when both peripheral and CHT-derived macrophages are depleted homogeneously. In contrast, macrophage recruitment to a wound is impaired by specific depletion of peripheral macrophages, revealing a specific role for these cells in the inflammatory response.

### A Reduced Peripheral Macrophage Pool Impairs Tail Fin Regeneration in Zebrafish Larvae

**Figure 4:**
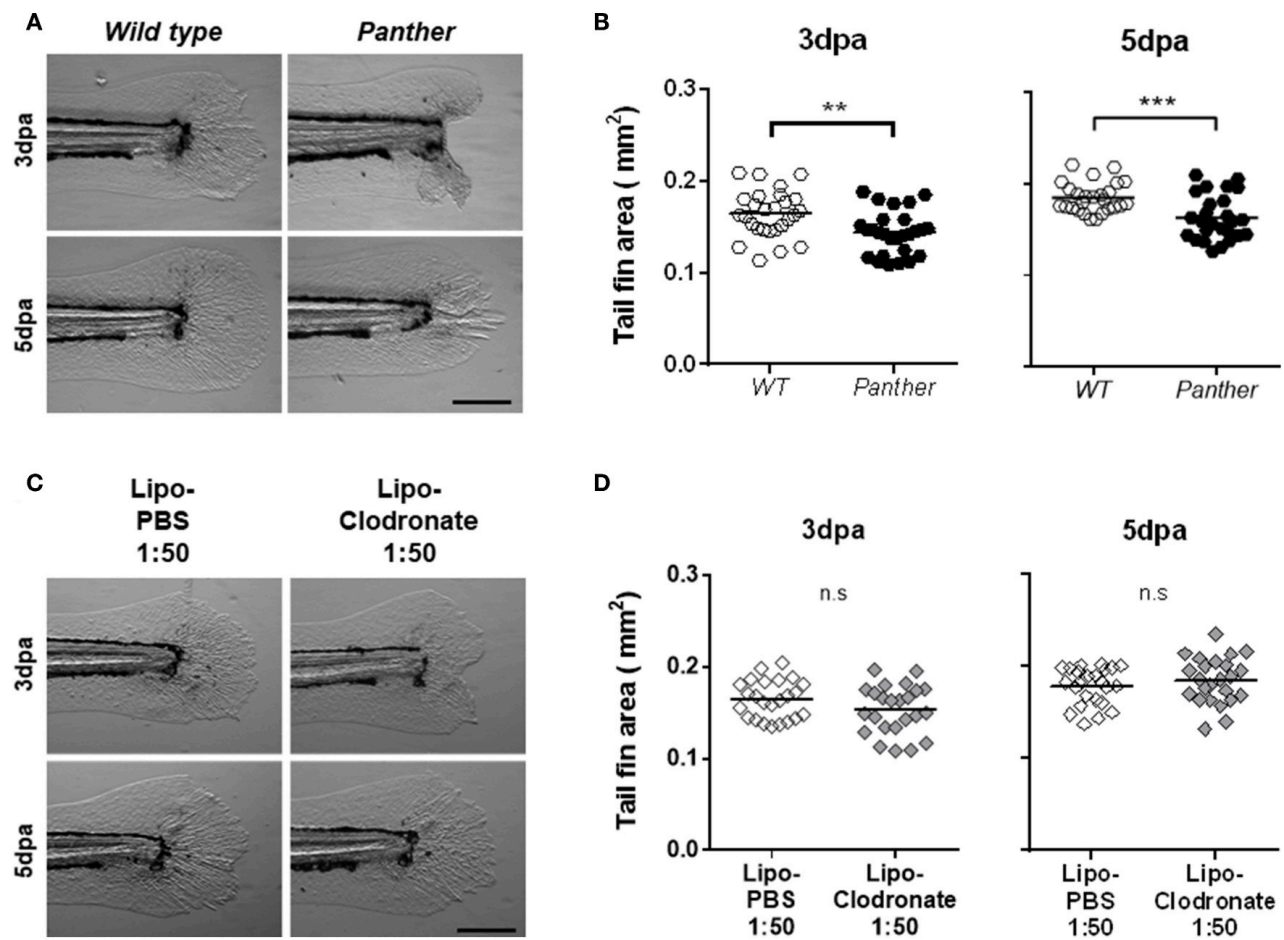
*Panther* individuals exhibit an impaired tail fin regeneration. The tail fin area of regenerating tail fins was calculated at 3 and 5 dpa. **(A)** Representative regenerating tail fins in *panther* and WT larvae. Scale bar = 200 μm. **(B)** Tail fin area quantification of regenerating tail fins in *panther* and WT larvae at both timepoints. A total of 27 larvae per group was used for the analysis. **(C)** Regenerating tail fin images of Lipo-clodronate 1:50 and Lipo-PBS 1:50 treated larvae. **(D)** Quantification of the tail fin area in Lipo-clodronate 1:50 and Lipo-PBS 1:50 treated individuals. A total of 24 larvae per group was used for the analysis. n.s not significant; ^**^p < 0.01; ^***^p < 0.001.

Previous studies have highlighted the importance of early recruited macrophages during zebrafish tail fin regeneration in both adult and larval stages (10, 11). We sought to determine if the impaired macrophage recruitment observed after tail fin amputation in *panther* mutants affects regeneration. We evaluated regeneration after amputation by measuring the tail fin area of regenerating fins in wild type and mutant 3 and 5 dpa fish. We found significantly impaired regeneration in *panther* mutants at both timepoints, when compared to wild type fish (Figures 4A,B). Again, as the number of macrophages is differentially affected in *panther* individuals depending on their place of residence in homeostasis (CHT vs. periphery), we wanted to determine if the regeneration phenotype in *panther* larvae is a consequence of the specific reduction in the peripheral tissue-resident macrophages or if it is due to the overall reduction of macrophages. We thus carried out tail fin amputation and regeneration analysis in 1:50 lipo-clodronate injected embryos and compared them to Lipo-PBS controls. As was the case for macrophage recruitment, we found no differences in regeneration efficiency between the lipo-clodronate and lipo-PBS conditions at 3 and 5 dpa (Figures 4C,D). Since *panther* mutants exhibit a strong reduction in the pool of peripheral macrophages (~60%; Figure 2C), we attempted to replicate this degree of reduction by injecting embryos with an increased concentration of lipo-clodronate. This effect was achieved by using a 1:10 dilution of lipo-clodronate; in injected fish, we observed a reduction in the peripheral macrophage pool similar to that of *panther* mutants (Supplemental Figures S3A–C). Under these conditions, we now observed impaired tail fin regeneration at 3 dpa in 1:10 lipo-clodronate injected fish compared to controls (Supplemental Figures S3D,E). Altogether, our results show that specifically decreasing the peripheral macrophage pool impairs tail fin regeneration after amputation in zebrafish larvae.

### An Increased Pro-inflammatory Environment and Reduced Cell Renewal Is Observed in *panther* Mutants After Tail Fin Amputation

**Figure 5:**
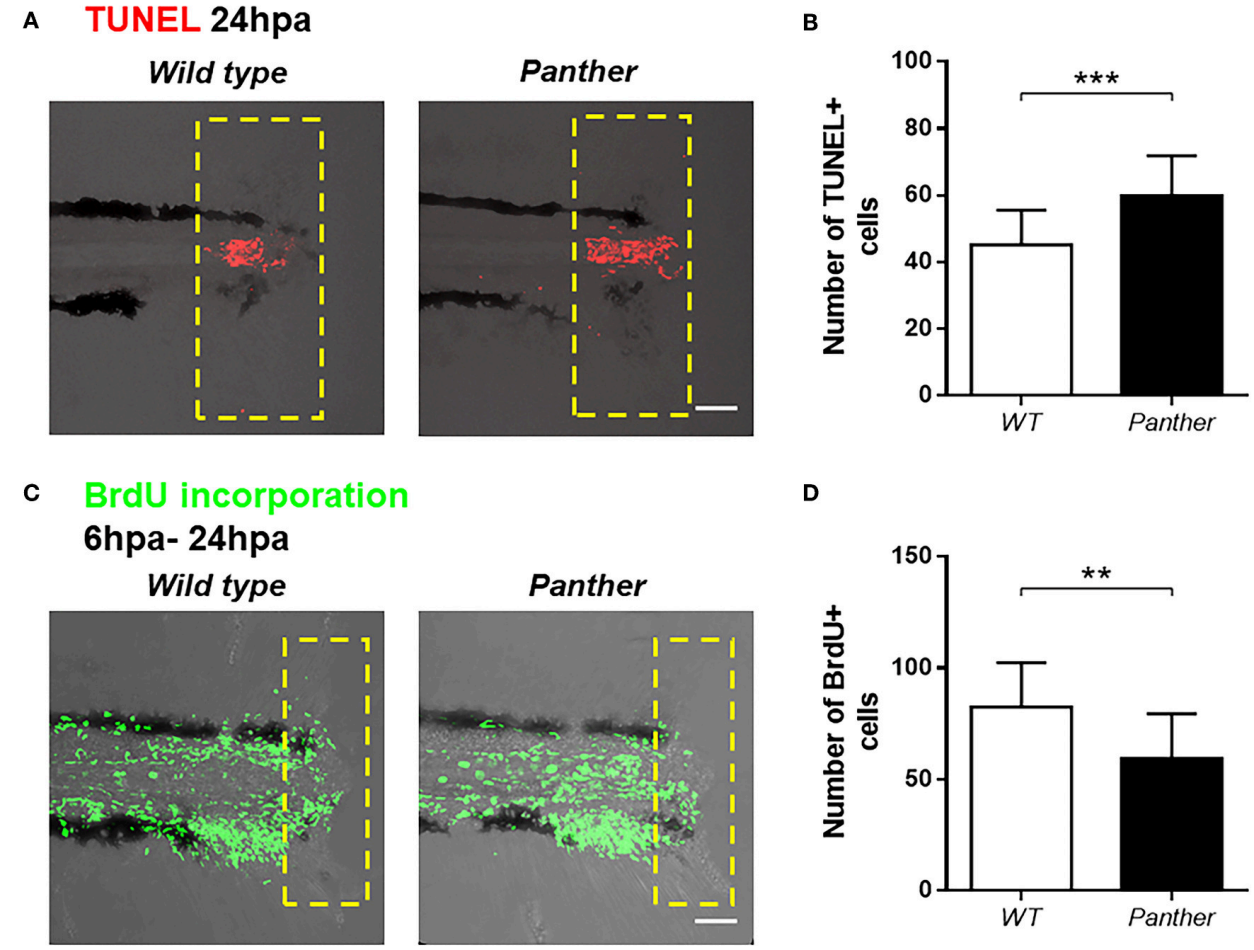
Increased cell death and reduced cell proliferation after tail fin amputation in *panther* larvae. **(A)** Cell death was measured at 24 hpa through TUNEL assays. Red dots represent TUNEL+ cells. **(B)** Quantification of TUNEL+ cells ± *SD* in the damage site of *panther* and WT larvae at 24 hpa. Twenty larvae per condition were used. **(C)** Cell proliferation was assessed by BrdU incorporation from 6 to 24 hpa. Green dots represent BrdU+ cells **(D)** The number of BrdU+ cells ± *SD* in *panther* and WT individuals was obtained from 12 larvae per condition. ^**^p < 0.01; ^***^p < 0.001.

To further characterize the impaired tail fin regeneration observed in *panther* mutants, we analyzed both cell death and cell proliferation after injury in these animals compared to wild type fish. We performed TUNEL staining at 24 hpa and we found a significant increase in the number of dead cells in *panther* mutants (Figures 5A,B). Furthermore, cell proliferation, measured by BrdU incorporation in the tail from 6 to 24 hpa, was reduced in *panther* mutants (Figures 5C,D). Thus, the impaired regeneration displayed by *panther* mutants can be explained by elevated cell death events and loss of proliferative ability.

**Figure 6:**
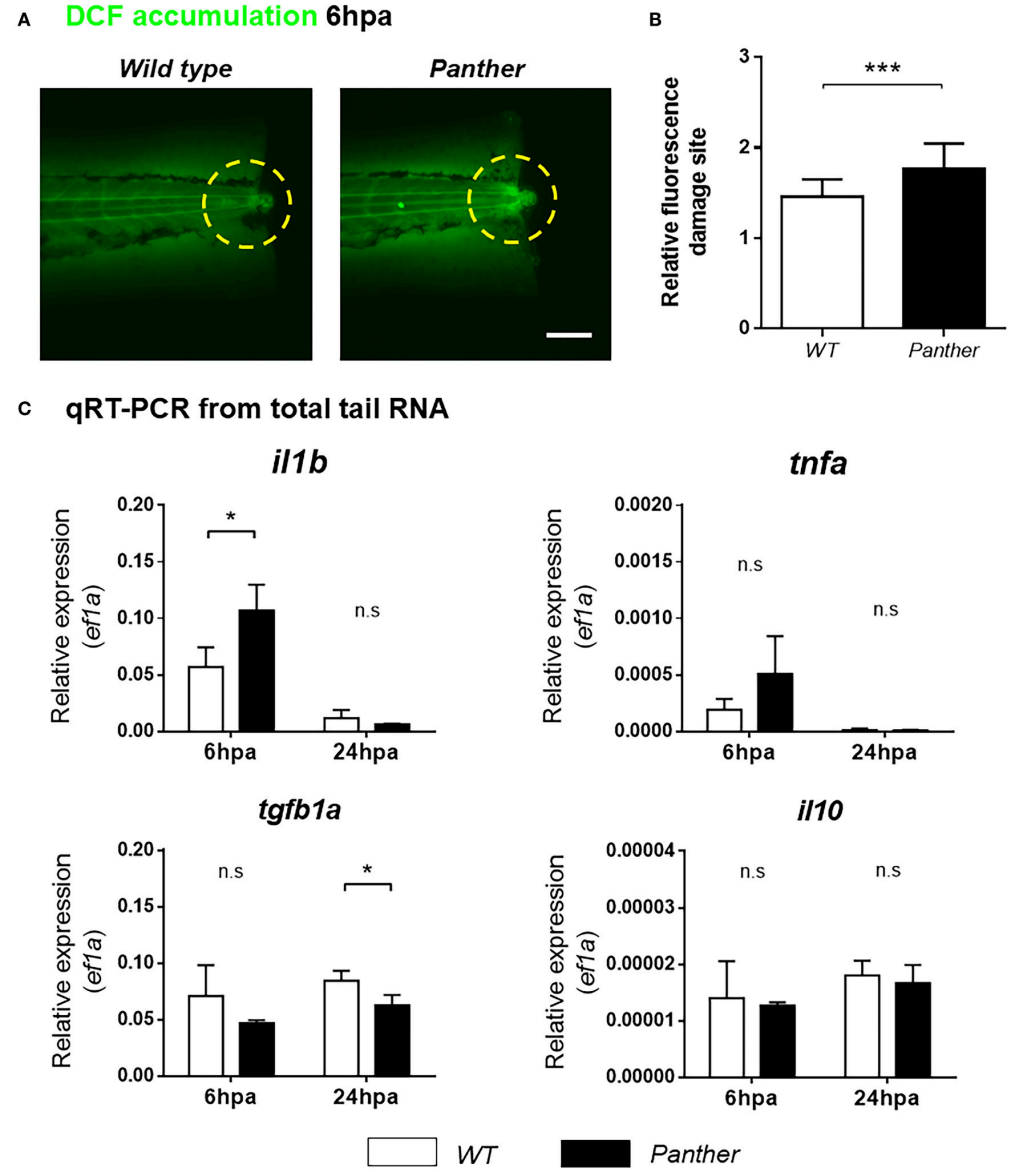
Heightened *il1b* expression and ROS in the damage site of *panther* larvae after tail fin amputation. **(A)** The amount of ROS generated in the damage site (yellow dashed circle) was measured through the accumulation of the fluorescent 2′7′-dichlorofluorescein (DCF) sensor. Scale bar = 100 μm. **(B)** Relative fluorescence in the damage site ± *SD* of *panther* and wild type larvae. Twenty larvae per condition were analyzed. **(C)** Quantitative RT-PCR for *il1b, tnfa, tgfb1a*, and *il10* from tails of *panther* and WT larvae at 6 and 24 hpa. The expression of *ef1a* was used as housekeeping for the 2^−Δ*Ct*^ calculation. A pool of ~20 larval tails was collected for RNA isolation, and the graphs show the mean ± *SD* of three independent experiments per condition. n.s not significant; *p < 0.05; ^***^p < 0.001.

Next, we sought to understand how recruited peripheral tissue-resident macrophages contribute to tissue regeneration. One of the initial responses after tissue damage is the production of ROS, which promote leukocyte recruitment (34), and potentiate the inflammatory response (35). To analyze ROS accumulation in the damage site, we used the sensor H_2_DCF-DA, which is catabolized to the fluorescent compound 2′7-dichlorofluorescein (DCF) through intracellular esterase activity and oxidation (36). We found that DCF accumulation in the damage site is higher in *panther* compared to wild type larvae at 6 hpa (Figures 6A,B), indicating that ROS production after damage is increased in *panther* animals.

New evidence indicates that pro-inflammatory cytokines play a major role during zebrafish tissue regeneration. Hasegawa et al. demonstrated that recruited macrophages downregulate *il1b* expression in the tissue during inflammation after amputation of the tail fin fold, thus controlling inflammation, decreasing cell death and promoting its regeneration (37). In a similar experimental setting, Nguyen-Chi et al. observed that the Tnfa/Tnfr1 signaling promotes macrophage recruitment and stromal proliferation, and that this factor is required for the regeneration of the tail fin fold (11). We analyzed the expression of both markers, *il1b* and *tnfa* mRNA, in the tail region using quantitative RT-PCR in *panther* and wild type animals. We found that *il1b* mRNA levels, but not *tnfa* mRNA, are increased in *panther* mutants at 6 hpa (Figure 6C). However, this difference in *il1b* expression became indistinguishable between *panther* and wild type at 24 hpa (Figure 6C). In mammals, macrophages suppress pro-inflammatory signaling through IL-10 and TGFβ (8, 9); hence, we sought to analyze the expression of these anti-inflammatory cytokines in our experimental setting. We did not find differences in *il10* expression after damage, but we observed an impaired *tgfb1a* expression in *panther* larvae at 24 hpa, when compared to wild type (Figure 6C). Our results show that *panther* mutants, which display decreased numbers of peripheral macrophages, exhibit an increased pro-inflammatory environment, and an impaired anti-inflammatory response after tail fin amputation, which correlates with a reduced proliferation and an increased cell death in the injured site (Figure 5), as well as impaired tail fin regeneration (Figures 4A,B). Therefore, we propose that peripheral macrophages contribute to tail fin regeneration through the negative regulation of the inflammatory environment in the damage site.

## Discussion

In mammals, macrophages are classified as a heterogeneous population of cells, with different origins and specific functions according to their location and activation status (14, 38). In zebrafish, recent evidence suggests a similar heterogeneity of macrophages during development (39, 40) and in different inflammatory settings, such as after tissue damage (19) and infection (41, 42). The lack of specific markers to identify different populations of macrophages in zebrafish, can be overcome using intravital imaging of fluorescently-labeled macrophages in the transparent larva. This feature has been previously described as a means to differentiate macrophages and microglia in the zebrafish retina (43). In this work, we used this advantage to classify macrophages according to their location in 3 dpf larvae as either peripheral- or CHT-resident, and to track them during homeostasis and during their recruitment to an injury site. We found that these populations have different speeds of migration before and after damage, with peripheral macrophages being faster than CHT macrophages in both conditions. The rapid response of peripheral macrophages could be attributed to an increased ability to sense danger signals when they reside in peripheral tissues, as a consequence of a differential maturation status, in line with a recent publication describing the importance of the *il34*-*csf1ra* axis in the colonization and maturation of macrophages in peripheral tissues such as CNS (31). Studies in mammals have shown that tissue-resident macrophages, that behave as sentinels distributed throughout the organism, can respond rapidly to early inflammatory signals, such as hydrogen peroxide (H_2_O_2_), calcium, ATP and alarmins (44). It is also known that the initial recruitment of zebrafish leukocytes to an inflammatory event is due to an H_2_O_2_ gradient generated rapidly after damage (34). Thus, we suggest that tissue-resident are more prone than CHT-resident macrophages to detect these signals and to migrate to the signal source. In line with this idea, it has been recently demonstrated that zebrafish tissue-resident macrophages are the first responders after *Mycobacterium marinum* infection in the hindbrain ventricle (42), thus emphasizing that peripheral tissue-resident macrophages and CHT-derived macrophages are distinct—at the very least—in their sensitivity to a variety of recruitment signals.

An important function of tissue-resident macrophages is to boost the recruitment of more macrophages and other immune cells, such as neutrophils (38). To analyze the contribution of zebrafish peripheral tissue-resident macrophages during the inflammatory response we used the previously described *panther* mutants. These fish have a macrophage phenotype in which there is a modest reduction in the CHT-resident population (~20%) and a severe reduction of the peripheral macrophage population (~60%) (16, 32). A similar phenotype has been described for the phosphate exporter mutant *xpr1b*, which also shows a reduced tissue-resident macrophage population (45). In *panther* mutant fish, the number of macrophages recruited to the injury caused by tail fin amputation is strongly reduced compared to wild type fish (31), an expected finding given the decrease in total number of macrophages. However, this decreased recruitment is not due merely to the overall macrophage reduction, as the effect is still significant after normalizing for the difference in total macrophages in mutant vs. wild type animals. Our interpretation for the result is that the selectively reduced pool of peripheral macrophages, that are recruited earlier to the injury site than their CHT-resident counterparts, are insufficient to secondarily recruit CHT-resident macrophages at 6 and 12 hpa. We provide support for this interpretation by depleting both peripheral and CHT macrophages in uniform fashion using lipo-clodronate at a 1:50 dilution (~40% reduction for both populations, which is similar to the overall decrease seen in *panther* mutants); in these fish, we did not see a decrease in recruitment after normalization. Therefore, *panther* mutants show a decreased migratory response to tissue damage not accounted for by the reduction in the number of macrophages but, rather, by the selective loss of the periphery-resident population. It is noteworthy that we did not observe differences in the recruitment of neutrophils either in *panther* mutants or in 1:50 lipo-clodronate injected fish, thus excluding a possible role of peripheral macrophages in the recruitment of neutrophils after tissue damage, in line with previous reports (10, 33).

As previous reports indicated that early recruited macrophages are important for proper tail fin regeneration in zebrafish larvae and adults (10, 11), we analyzed the regeneration of the tail fin after amputation in *panther* larvae. We found that tail fin regeneration is delayed in *panther* mutants at 3 and 5 dpa, compared to wild type fish. Again, we were able to exclude that the regeneration phenotype observed in *panther* mutants is a consequence of the overall reduction in the macrophage number in the larvae. To achieve this, we phenocopied the *panther* phenotype by treating fish with 1:50 lipo-clodronate in order to reduce the overall number of macrophages to a similar extent (~40% reduction). In these animals, we did not observe differences in the tail fin area at 3 and 5 dpa, indicating that the impaired regeneration observed in *panther* mutants is due to the specific reduction of the peripheral macrophage pool. Further, depletion of peripheral macrophages leads to reduced cell proliferation, increased cell death and a pro-inflammatory environment, manifested by the increased ROS and *il1b* expression in the damage site.

New insights have revealed that inflammation is a necessary step to trigger the regeneration response (46), and ROS is one of the first pro-inflammatory signals produced after tissue damage. Previous work has demonstrated that ROS can act as signaling cues that favor the expression of pro-regeneration genes (47, 48). However, sustained ROS production can lead to cell death and chronic inflammation (35), so its activity needs to be finely regulated. The increased DCF staining observed in *panther* larvae suggest that peripheral macrophages may contribute to downregulate ROS accumulation at the damage site. Although is not clear how macrophages contribute to ROS downregulation during an inflammatory response, we think this role might be performed directly by macrophages, through the expression of cytoprotective molecules (49, 50), or indirectly through the regulation of other cells such as neutrophils, that can negatively regulate the H_2_O_2_ generated immediately after tissue damage through the expression of myeloperoxidase (51). Further studies are needed to provide a mechanism underlying ROS regulation by macrophages.

In addition to ROS, the activity of *il1b* and *tnfa* could be relevant to understand the impaired tail fin regeneration phenotype observed in *panther* mutants. In zebrafish, it has been previously shown that transient *il1b* expression promotes the upregulation of regeneration-related trophic factors required for fin fold regeneration, although prolonged expression of *il1b* leads to aberrant apoptosis and impaired regeneration (37). On the other hand, proliferation of stromal cells depends on Tnfa/Tnfr1 signaling, with macrophages being the cells that release Tnfa in the damaged area (11). Since we observed increased expression of *il1b* in *panther* mutants at 6 hpa, and no change in *tnfa* RNA levels at the same timepoint (Figure 6C), we hypothesize that peripheral tissue-resident macrophages contribute to down-regulate *il1b* expression in the tissue during the early response to damage, rather than promoting expression of *tnfa*. A recent report has shown that macrophages isolated from *panther* larvae are less efficient in inducing *tnfa* expression after blood vessel damage, but this impairment can be reversed through the administration of pro-inflammatory stimulants (52). As tail fin amputation in *panther* larvae triggers a strong pro-inflammatory response, we think that *tnfa* expression by recruited macrophages might not be affected in this experimental setting, as they can sense an inflammatory environment. Moreover, it is possible that, in a normal inflammatory event, *il1b* and *tnfa* may be expressed at different times, with *il1b* acting in an early period and macrophage-derived *tnfa* acting as a response to decreasing *il1b*, itself downregulated by peripheral macrophages. This sequence of events could be needed to recruit cells that can dampen the inflammatory phase and transition into stromal cell proliferation and tissue regeneration. Hence, if both molecules were to be expressed at the same time, they could prolong a pro-inflammatory microenvironment that increases oxidative stress and cell death, leading to delayed or impaired regeneration of the tail fin. This unfavorable outcome could be significant even if the levels of both *il1b* and *tnfa* levels are downregulated at a later period. The persistent co-expression of IL-1β and TNFα has been described in different pathological conditions, where it perpetuates cell death and tissue destruction (53, 54). Further information is required to understand the kinetics of *il1b* and *tnfa* expression during inflammation and regeneration, as well as their possible link with the described macrophage populations. In this sense, the reporter line *Tg(tnfa:GFP)* (19) would be useful to determine if peripheral macrophages acquire an M1–M2 profile when recruited to the damage site.

It is known that mammalian macrophages produce IL-10 and TGFβ to downregulate pro-inflammatory signals after tissue damage, in order to avoid excessive inflammation that negatively impact on tissue repair or regeneration (8, 9). From these anti-inflammatory cytokines, we only observed a reduction in *tgfb1a* expression at 24 hpa in *panther* larvae, when compared to wild type (Figure 6C). Although the timepoint of reduced *tgfb1a* expression (24 hpa) is not coincident with the increased *il1b* expression (6 hpa), our results suggest that peripheral macrophages promote *tgfb1a* expression in the damage site, thus modulating pro-inflammatory signaling. Previous studies have shown that Tgfb/Activin pathway, including *tgfb1*, is active in blastema cells during regeneration in zebrafish and amphibians, promoting its proliferation (55–58). Therefore, the reduced *tgfb1a* expression could also be a consequence of the impaired blastema proliferation, and not only due to the lower recruitment of peripheral macrophages. A more detailed analysis of macrophages recruited to the damage site is required to better understand the relationship between these cells and *tgb1a* expression, as well as other components of the Tgfb/Activin pathway, in the control of the inflammatory response and tissue regeneration.

We conclude that the inflammatory microenvironment triggered by the amputation of the tail fin is fine-tuned mainly by macrophages that reside in peripheral tissues, and that are recruited during early time points after tissue damage. The outcome of the arrival dynamics and interplay between peripheral tissue-resident and CHT-resident macrophages strongly suggests functional differences between these two groups of macrophages in zebrafish, as well as specific roles during immune responses.

## Author Contributions

RM conceived the idea, designed and performed experiments, and wrote the draft manuscript. MA participated in the experimental design and generated the final manuscript. RM and MA read, edited, and approved the final manuscript.

### Conflict of Interest Statement

The authors declare that the research was conducted in the absence of any commercial or financial relationships that could be construed as a potential conflict of interest.

## Abbreviations

BrdU: 5-Bromo-2′-deoxyuridine
CHT: Caudal hematopoietic tissue
*csf1ra*: Colony stimulating factor 1 receptor a gene
DCF: 2′7′-dichlorofluorescein
dpa: days post amputation
dpf: days post fertilization
H_2_O_2_: hydrogen peroxide
*il10*: interleukin 10
*il1b*: interleukin 1b
hpa: hours post amputation
hpf: hours post fertilization
Lipo-clodronate: Clodronate-encapsulated liposomes
Lipo-PBS: PBS-loaded liposomes
Mϕ: Macrophage
qRT-PCR: Quantitative reverse transcription polymerase chain reaction
ROS: reactive oxygen species
SD: standard deviation
*tgfb1a*: transforming growth factor b1a
*tnfa*: tumor necrosis factor a
TUNEL: Terminal deoxynucleotidyl transferase dUTP nick end labeling
WT: wild type.
