# Supplementary material for: Peripheral Macrophages Promote Tissue Regeneration in Zebrafish by Fine-Tuning the Inflammatory Response
Source: Front Immunol. 2019 Feb 15;10:253. doi: 10.3389/fimmu.2019.00253 (PMC6413720; doi:10.3389/fimmu.2019.00253)
Supplement: Supplementary file 1 [file Table_1.DOCX]

**Supplemental Figures**


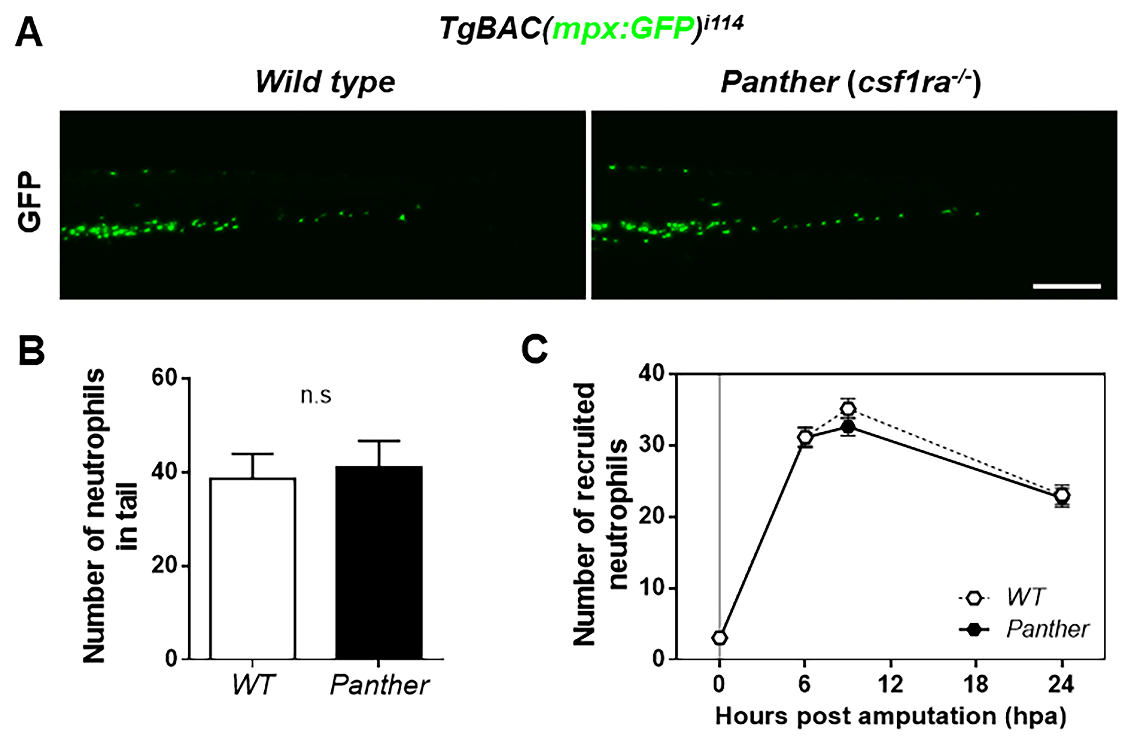


**Figure S1. Neutrophil number and their recruitment are not affected in *panther* larvae.** (A) Representative tail images of 72hpf *Tg(mpx:GFP)^i114^* neutrophil reporter larvae in wild type and *panther* genetic background. Scale bar = 250 µm. (B) Total number of neutrophils ± SD in the tail of *panther* and wild type larvae. n = 6-7 larvae per condition. (C) Quantification of recruited neutrophils after tail fin amputation ± SEM in *panther* (black solid line) and wild type (dashed line) larvae from 0hpa to 24hpa. 20 larvae per condition were used for the analysis. n.s not significant difference.


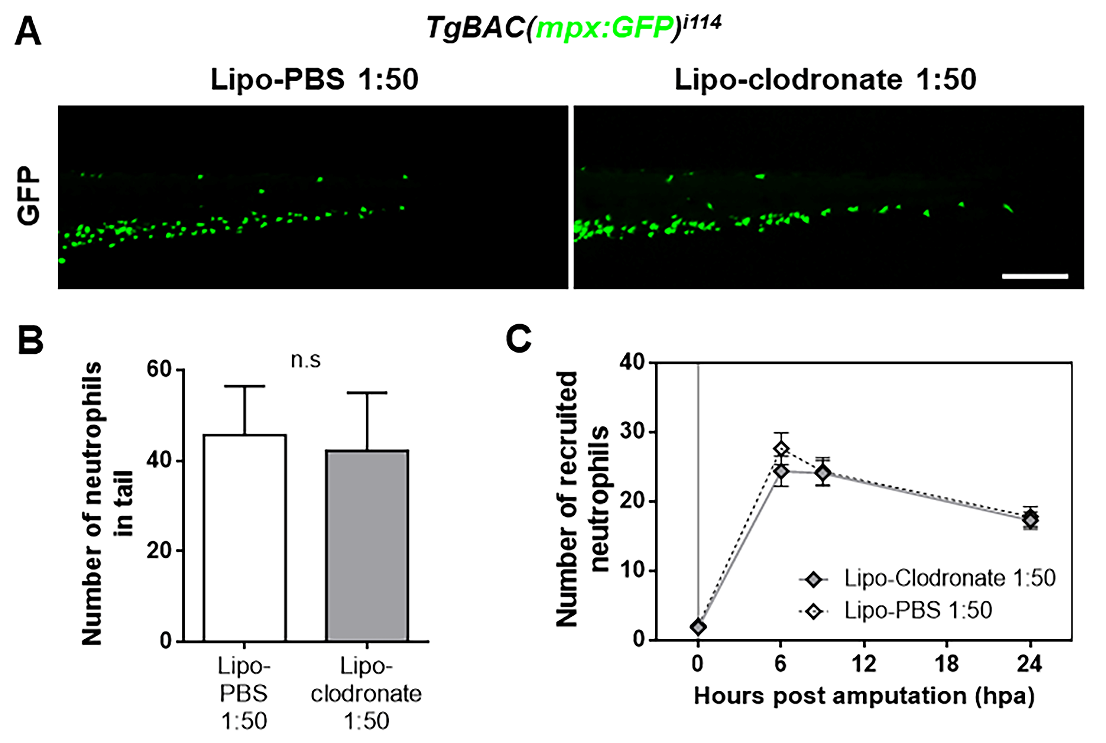


**Figure S2. Injection of Lipo-clodronate 1:50 does not affect neutrophil population in zebrafish larvae.** (A) Representative tail images of 72hpf *Tg(mpx:GFP)^i114^*, 18 hours post injection of Lipo-clodronate 1:50 and Lipo-PBS 1:50. No differences in the density or the distribution of neutrophils were observed. Scale bar = 250 µm. (B) Mean ± SD of total neutrophils in the tail of Lipo-clodronate 1:50 and Lipo-PBS 1:50 at 72hpf. 20 larvae per condition were analysed. (C) Recruitment of neutrophils in Lipo-clodronate 1:50 (gray solid line) and Lipo-PBS 1:50 (dashed line) larvae from 0hpa to 24hpa. 20 larvae per condition were used for the analysis. n.s not significant difference.


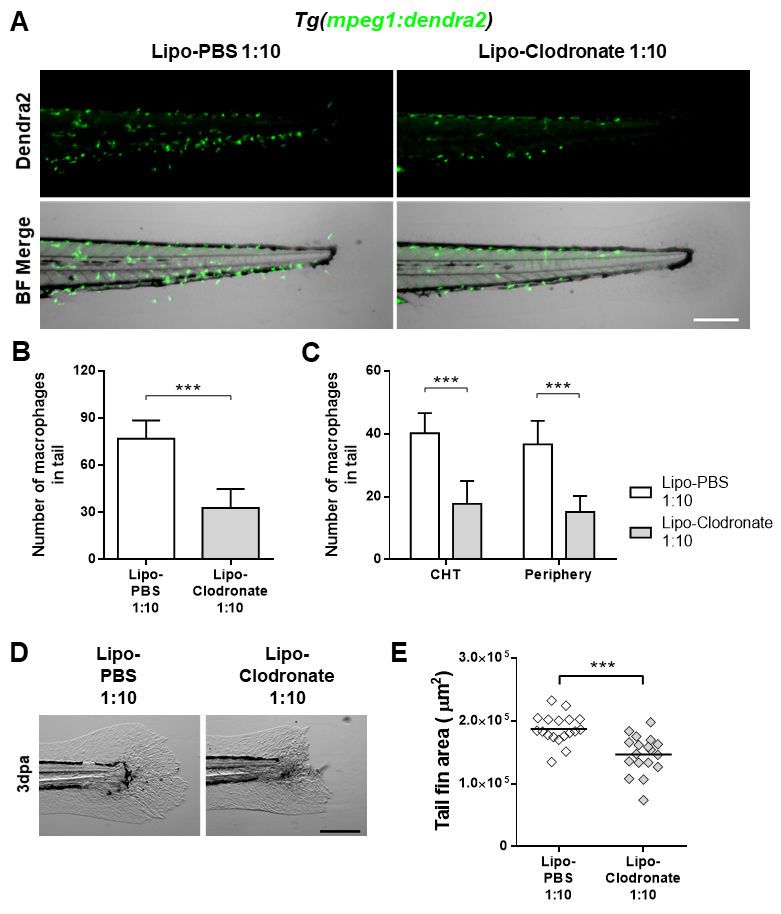


**Figure S3. Injection of Lipo-clodronate 1:10 severely reduces macrophage number in zebrafish larva and impairs tail fin regeneration after amputation.** (A) Representative images of 72hpf *Tg(mpeg1:Dendra2)* larvae after 18 hours of Lipo-clodronate 1:10 and Lipo-PBS 1:10 injections in the bloodstream. Scale bar = 250µm. (B) Number of total macrophages ± SD in the tail of Lipo-PBS 1:10 and Lipo-clodronate 1:10. (C) Quantification ± SD of peripheral tissue-resident and CHT-resident macrophages in the previously mentioned conditions. (D) Images of regenerating tail fins from Lipo-PBS 1:10 and Lipo-clodronate 1:10 larvae at 3 days post amputation. Scale bar = 200µm. (E) Quantification of the tail fin area in Lipo-PBS 1:10 and Lipo-clodronate 1:10 treated larvae at 3dpa. 20 larvae per condition were analyzed. *** p < 0.001.

**Supplemental Video information**

**Supplemental Video 1. Individual tracking of photoconverted macrophages recruited to the damage site after tail fin amputation in zebrafish larva.** Macrophages located in the CHT (A) or in the periphery (B) of *Tg(mpeg1:Dendra2)* larva were photoconverted at 72hpf, and then the tail fin was amputated to analyze its recruitment to the damage site. The complete tail of each selected larva was imaged every 5 minutes, from 30 minutes to 24 hours after tail fin amputation. The CHT is indicated in yellow at the beginning of the video. The damage site is shown as the region between the 2 yellow lines (~150µm between both lines). In the upper-left section of each video, the time is shown in a hour:minute format. Macrophage tracks are shown in blue. Scale bar = 100µm.
